# Robotic proctocolectomy with ileal pouch-anal anastomosis: a hybrid approach

**DOI:** 10.1007/s00384-025-04854-5

**Published:** 2025-03-12

**Authors:** Pietro Calabrese, Orsalia Mangana, Carlo Alberto Manzo, Laura Muirhead, Valerio Celentano

**Affiliations:** 1 Department of General Surgery, Transplantation and Gastroenterology, Federico II University Hospital, Naples, Italy; 2 Chelsea and Westminster Hospital NHS Foundation Trust, London, UK; 3 Division of Surgery and Cancer, Imperial College London, London, UK; 4 Division of General and HPB Surgery, Rho Memorial Hospital, ASST Rhodense, Rho, Milano Italy

**Keywords:** Robotic surgery, Proctocolectomy, Ileal pouch-anal anastomosis (IPAA), Hybrid approach

## Abstract

**Purpose:**

Proctocolectomy with ileal pouch-anal anastomosis (IPAA) is the definitive surgical treatment for patients with ulcerative colitis or familial adenomatous polyposis. While laparoscopic surgery has been widely adopted, robotic surgery has emerged as a promising alternative, particularly for complex pelvic dissection. However, the robotic learning curve presents significant challenges. This study introduces a hybrid approach combining robotic and laparoscopic techniques to leverage the strengths of both, with a focus on the impact of the robotic learning curve and surgical training.

**Methods:**

All patients undergoing hybrid or laparoscopic proctocolectomy with IPAA for ulcerative colitis between 2022 and 2024 were included. Data on patient characteristics and postoperative outcomes were collected prospectively. Primary outcomes were operating time and 30-day morbidity. All robotic procedures were performed by a consultant surgeon within their first 100 robotic cases.

**Results:**

A total of 25 patients were included: 14 in the hybrid group and 11 in the laparoscopic group. The median operating time was 300 min for the hybrid approach versus 325 min for the laparoscopic approach. In the hybrid surgery group, between 72 and 90% of the laparoscopic part of the procedure was performed by a supervised surgical trainee.

**Conclusions:**

The hybrid robotic-laparoscopic approach offers potential benefits over pure laparoscopy by reducing operative time and postoperative complications. Additionally, it provides a structured modular training opportunity, allowing surgeons to develop both laparoscopic and robotic skills, particularly during the early stages of their robotic learning curve.

## Introduction

Proctocolectomy with ileal pouch-anal anastomosis (IPAA) stands as a definitive surgical treatment for selected patients with ulcerative colitis and familial adenomatous polyposis. Traditionally performed via open or laparoscopic methods, restorative proctocolectomy involves the removal of the colon and rectum, followed by the creation of an ileal pouch to maintain intestinal continuity. IPAA is a complex and technically challenging procedure due to the intricacies involved in rectal dissection, appropriate level of rectal cuff transection, and the creation of a tension free anastomosis.[1] Mucosectomy is primarily indicated in IPAA to remove residual rectal mucosa, with hand-sewn pouch-anal anastomosis. Originally advocated to reduce the risk of dysplasia or cancer, its routine use remains debated due to potential impacts on continence and pouch function. The decision to perform mucosectomy should balance oncological safety with functional outcomes, particularly in patients at low risk of neoplastic transformation.[2] Stapled IPAA anastomosis is the most common approach nowadays.

Over recent years, robotic surgery has emerged as an innovative alternative to the laparoscopic approach for several colorectal procedures, offering enhanced and three-dimensional visualization, and greater dexterity, particularly in the deep pelvic dissection.[3] However, robotic-assisted surgery presents its own set of challenges with respect to the learning curve, operating time, and surgeon proficiency.

We aim to present a hybrid approach for robotic proctocolectomy with IPAA, which combines robotic pelvic surgery with selected laparoscopic steps, as a way to leverage the strengths of each technique while minimizing their respective weaknesses, particularly during the robotic surgery learning curve. We present a review existing literature on the operating times, complications, and outcomes of robotic proctocolectomy compared to laparoscopic approach, with a particular focus on the impact of the learning curve and surgeon experience. Finally, we discuss the introduction our refined hybrid technique, outlining its advantages in terms of reduced operative time, lower complication rates, and its suitability for surgeons during their learning curve and modular surgical training.

## Methods

This study was conducted in accordance with the STROBE (Strengthening the Reporting of Observational Studies in Epidemiology) checklist[4] to ensure the quality and transparency of reporting. The study was conducted in accordance with the principles of the Declaration of Helsinki[5] and the guidelines of Good Clinical Practice.[6]

All consecutive patients undergoing laparoscopic, robotic, or hybrid proctocolectomy with ileoanal pouch formation for ulcerative colitis during a 2-year study period from October 2022 to September 2024 were included. Patients under the age of 18, or patients undergoing 3 stage surgery were excluded, as were patients with Crohn’s disease, indeterminate colitis, or FAP. All included procedures were performed in 2 stages, with a covering loop ileostomy reversed 3 to 6 months after surgery, following water contrast enema x-ray or MRI pelvis, and pouchoscopy.

Baseline patients’ characteristics were collected prospectively, on an institutional approved database. Postoperative outcomes were collected for 30 days. Primary outcomes were 30-day morbidity and operating time. Secondary outcomes were conversion to open surgery, and modules of the procedures performed by trainees. Postoperative complications were recorded according to the Dindo-Clavien classification.[7]

The panproctocolectomy procedure was sub-divided into 3 different modules (proctectomy, left hemicolectomy, and right hemicolectomy) for the purpose of annotating the grade of the surgeon performing that particular module, if a consultant surgeon or a trainee,[8] annotating the video of the procedures accordingly.[9]

All robotic procedures were performed by a consultant surgeon during the first 100 cases of the robotic surgery learning curve.

Categorical variables are presented as frequency or percentage and were compared with the use of the chi-square test or Fisher’s exact test, as appropriate. Continuous variables are presented as mean (± standard deviation) or median (range) and were compared with the use of Student’s *t* test. The Mann–Whitney *U* test was used for continuous, not normally distributed outcomes.

Statistical analysis was performed by using the Statistical Package for Social Sciences (SPSS version 16.0; SPSS, Chicago, IL, USA). All reported *P* values were two-tailed, and *P* values of less than 0.05 were considered to indicate statistical significance. Informed consent was obtained from the patients.

### Hybrid approach for robotic proctocolectomy with IPAA

#### Stage 1: trocars position and robot docking

**Fig. 1:**
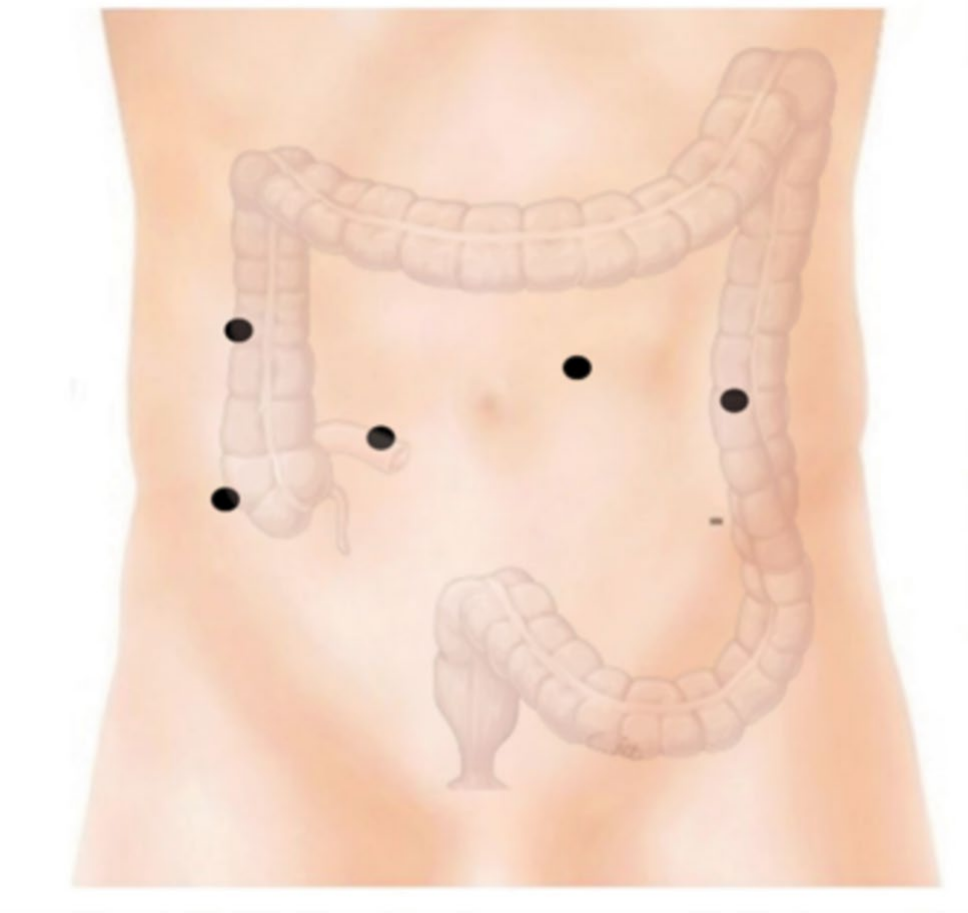
Position of the operating trocars

The procedure starts by accessing the peritoneal cavity at the site of the planned loop ileostomy, with a 12-mm robotic trocar via a wound protector. Three additional 8-mm robotic trocars are placed for robotic docking, targeting to the pelvis. An accessory 5-mm assistant trocar is inserted in the right upper quadrant, as demonstrated in Fig. 1, 8-cm distance is ensured between the robotic trocars, and the Da Vinci Xi system is docked. The site for the loop ileostomy is preoperatively marked by the stoma care team.

### Stage 2: robotic anterior resection with total mesorectal excision (TME)

**Fig. 2:**
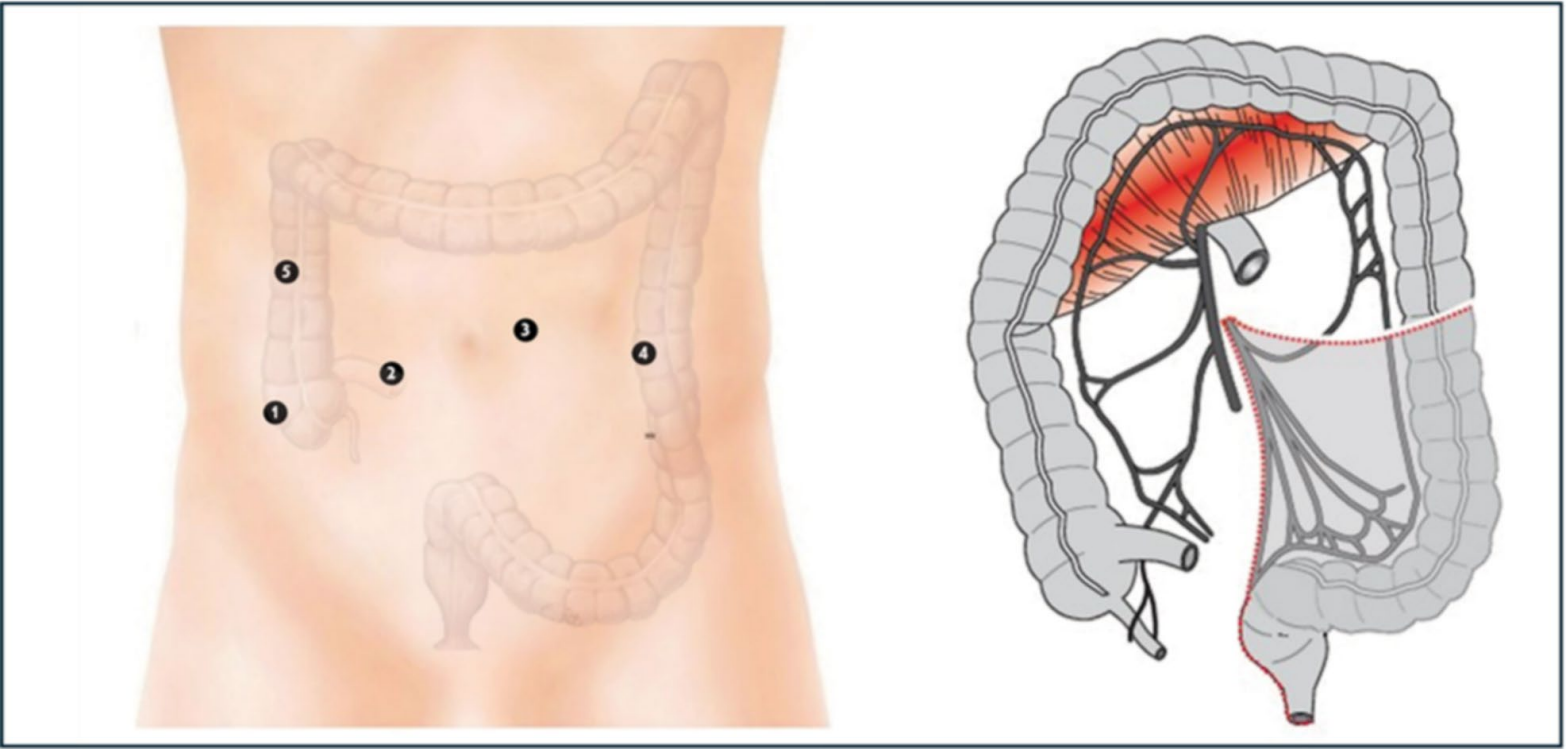
Robotic proctectomy: therobotic camera is placed in trocar #3, while robot arms are connected to trocars #1, #2, and #4. Trocar #5 is used by the assistant

The robotic phase involves ligation of the inferior mesenteric artery (IMA) and a total mesorectal excision, extending the rectal dissection down to the level of the levator ani muscles, where the rectum is transected with a robotic stapler, following repeated digital rectal examination to ensure correct height (Fig. 2).

This step is crucial in the context of ulcerative colitis, minimizing risks of recurrent cuffitis and anastomotic strictures while preserving functional outcomes. The sigmoid colon is mobilized laterally as much as it is allowed by the trocar positioning. In benign proctectomies, we follow the TME plane posteriorly and anteriorly, as more anatomical and bloodless, while anteriorly we dissect close to the rectum, to minimize risk of urogenital dysfunction, in a “near-TME” plane.[10]

### Stage 3: robot undocking and laparoscopic assisted colectomy

**Fig. 3:**
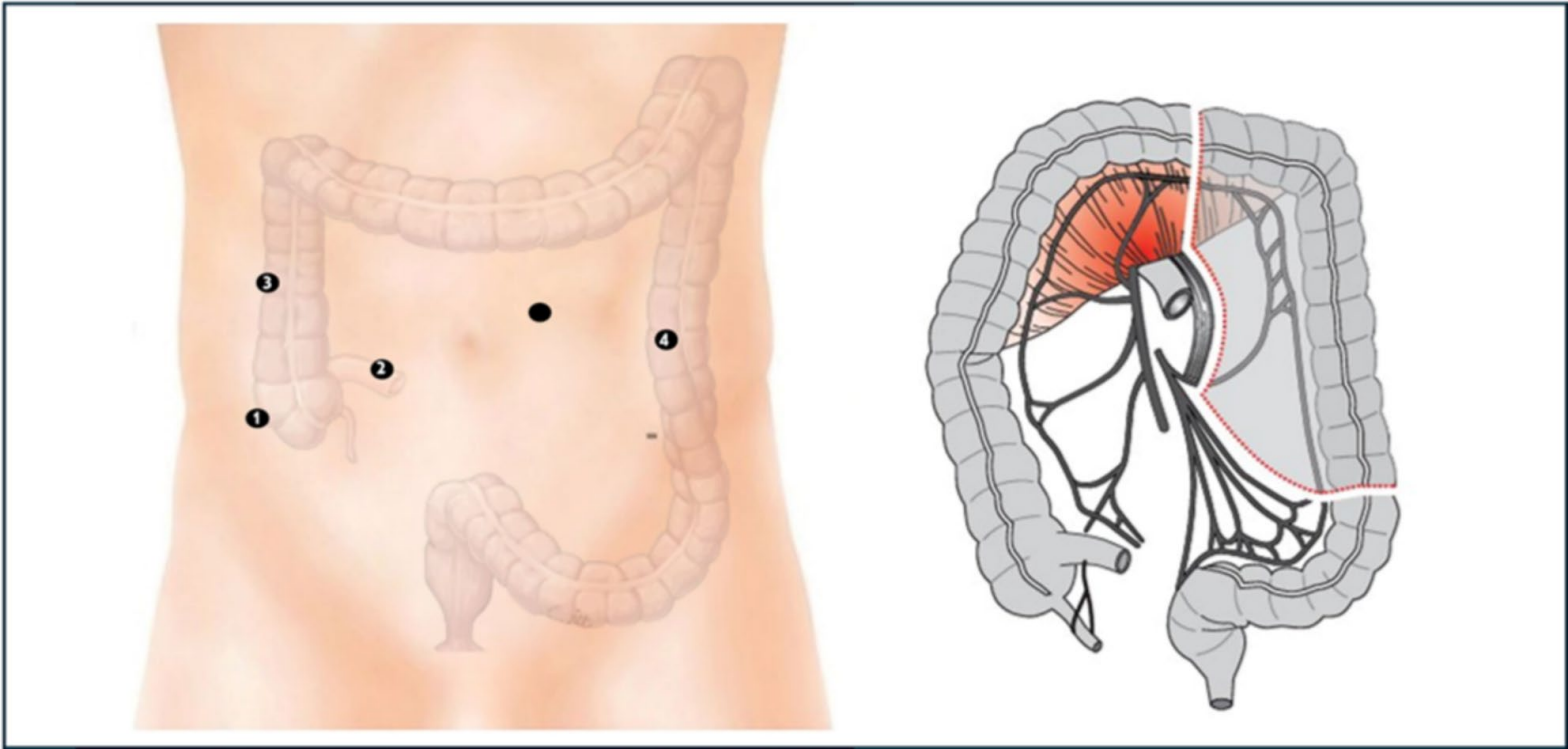
Laparoscopic left colectomy: thesurgeon is standing on the right side of the patient using operating trocars #1 and #3,while the assistant is retracting using trocar #4. The camera can be moved into trocar #2 or periumbilical.The reverseTrendelenburg position can facilitate this part of the procedure

Upon completion of the robotic module, the procedure transitions into the laparoscopic phase, using the same trocar sites (Fig. 3), with surgeon and assistant standing on the patient’s right side. The mobilization of the splenic flexure and isolation of the inferior mesenteric vein (IMV) are completed. The division of the mesocolon requires an energy device during the laparoscopic phase.

**Fig. 4:**
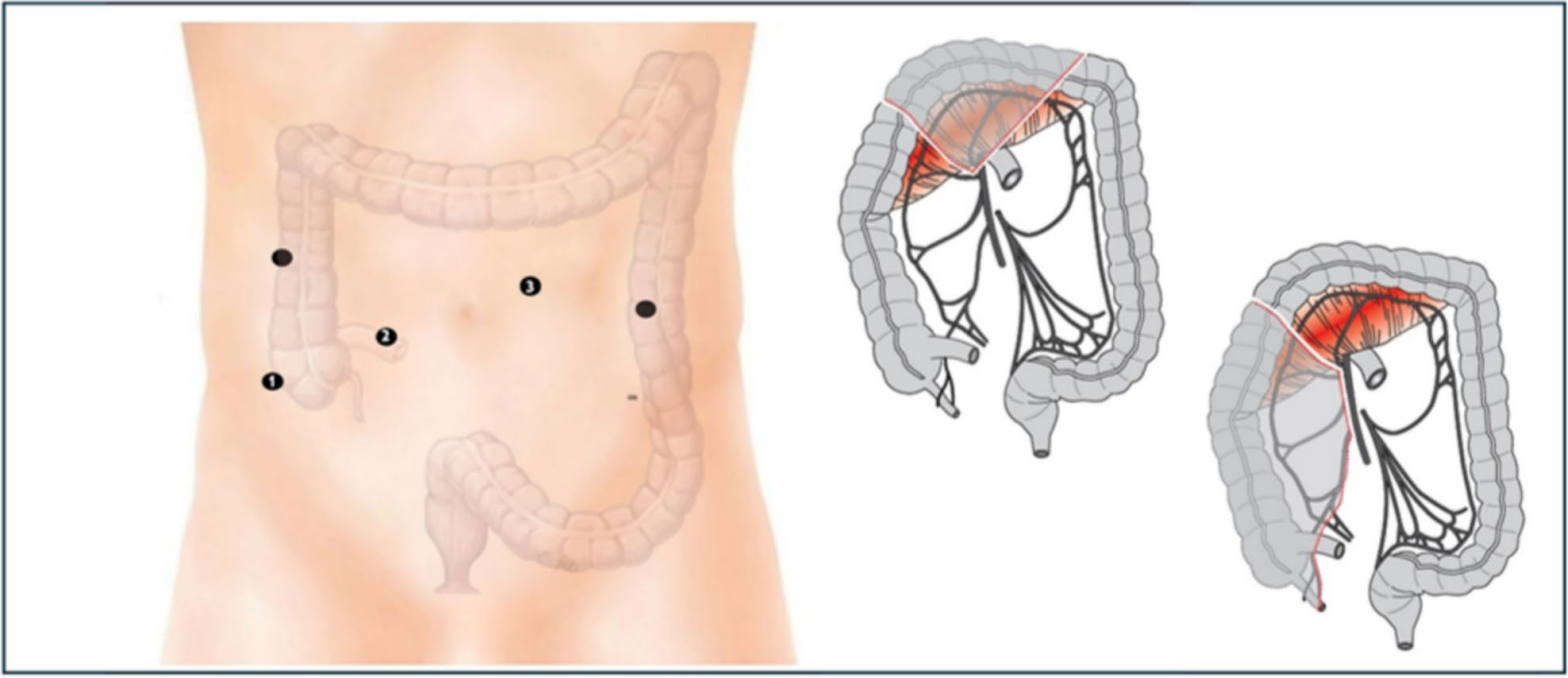
Laparoscopic colectomy of the transverse and right colon: thesurgeon positions themselves between thepatient’s legs or to the left. Trocar #2 is designated for the laparoscopic camera, while trocars #1 and #3 are for operating instruments. The remaining trocars can be use by the assistant. If needed, the camera can be inserted into trocar #3, allowing the surgeon to choose the most appropriate and ergonomic operating trocars based on the patient’s anatomy

The surgeon then moves on the right side of the patients or between the legs to progress the laparoscopic dissection of the transverse colon, extending the opening of the gastro-colic ligament and controlling the middle colic vessels. Subsequently, the right colectomy is completed with high ligation of the ileocolic pedicle following a sub-ileal approach, critical to ensure tension-free anastomosis (Fig. 4).

### Stage 4: fashioning of the J-pouch

We routinely perform this step extracorporeally.[11] A trial descent to the pelvic floor of the presumed pouch reservoir is completed laparoscopically prior to specimen extraction to ensure enough length is present for tension-free anastomosis. The specimen is extracted via a transverse supra-pubic incision, or preferably at the site of the planned loop ileostomy site. Following transection of the terminal ileum, the ileal pouch is created extracorporeally with a stapled technique. Relaxing mesenteric incisions and lengthening manoeuvres are performed as required.

### Stage 5: re-docking of robot and ileal pouch-anal anastomosis

Once the pouch is created, the robotic system is re-docked to perform an ileoanal anastomosis, ensuring optimal alignment of the small bowel mesentery and vascularization. An on-table pouchoscopy is routinely performed, predominantly to check for bleeding and leaks.

**Fig. 5:**
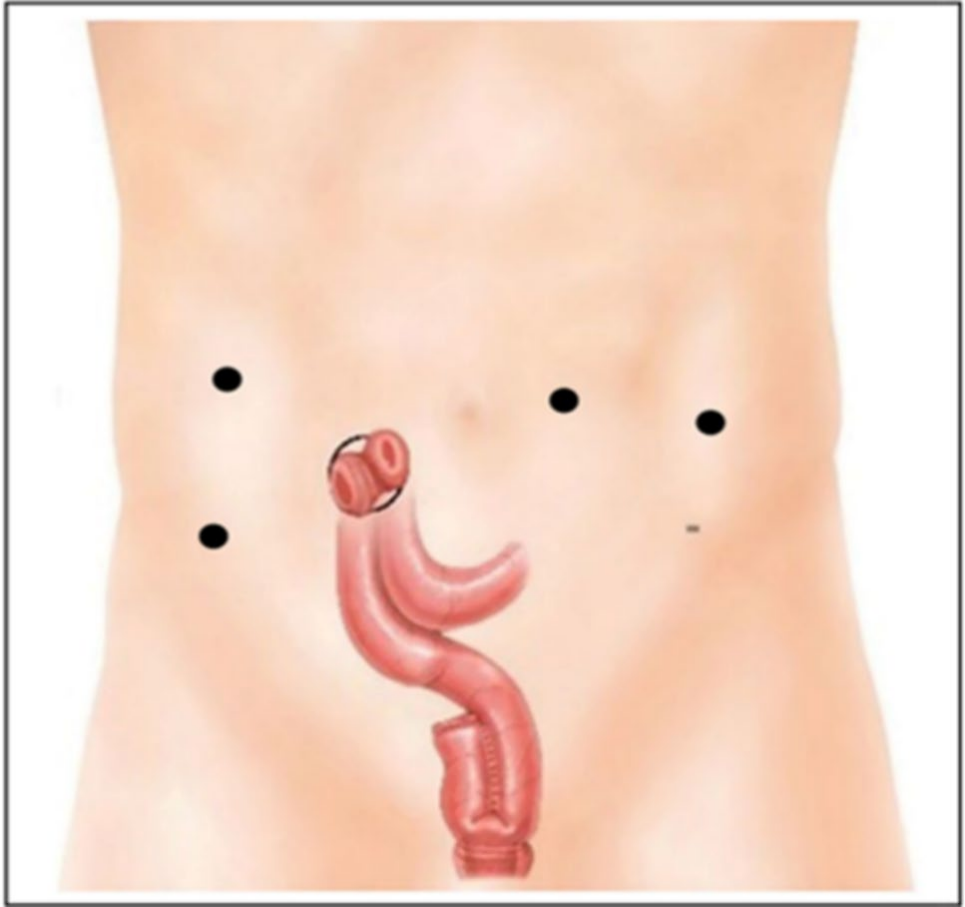
Final overview: trocar#3 is used for the protective ileostomy site

The procedure concludes with the formation of the loop ileostomy (Fig. 5).

These steps can also be performed with a pure laparoscopic approach.

## Results

Then, 14 patients underwent 2-stage restorative proctocolectomy with the hybrid laparoscopic and robotic approach, while 11 patients underwent a pure laparoscopic procedure.

Patients’ baseline characteristics, indication for surgery, and postoperative outcomes are illustrated in Table 1.

**Table 1:** Patients’ baseline and postoperative outcomes

	Hybrid robotic/laparoscopic (*n* = 14)	Laparoscopic only (*n* = 11)
Age	36 (28–57)	31 (19–51)
Gender (M: F)	8: 6	6: 5
Indication for surgery	Ulcerative Colitis: 14 (100%) - Medically refractory disease: 11 (78.57%) - High grade dysplasia: 2 (14.29%) - Adenocarcinoma: 1 (7.14%)	Ulcerative colitis: 11 (100%) - Medically refractory disease: 9 (81.82%) - High grade dysplasia: 2 (18.18%)
Stages of IPAA	2-stage: 14 (100%)	2-stage: 11 (100%)
Body mass index (BMI)	26 (23–30)	25 (22–29)
Conversion to open	0	0
Operating time (min)	300 (225–480)	325 (240–510)
Reoperation	0	1 (9.09%) Small bowel obstruction with refashioning of stoma
30-day morbidity overall	1 (7.14%) Prolonged post-op ileus	4 (36.36%) 1 prolonged ileus, 2 collections, 1 reoperation
30-day morbidity (grade ≥ 3 Clavien-Dindo)	0	3 (27.27%) 1 reoperation, 2 collection
Readmissions	0	2 (18.18%) 1 ileus treated conservatively, 1 postoperative collection requiring drainage

None of the results reached statistical significance (<0.05)

### Modular training

The hybrid robotic and laparoscopic approach involved completion of the proctectomy by the consultant surgeon in all 14 cases. However, the laparoscopic approach to right colectomy and left hemicolectomy was performed by a supervised trainee in 13 (92.8%) and 10 (71.4%) of the hybrid cases, respectively, similarly to the laparoscopic group where the laparoscopic right colectomy and left colectomy was performed in 9 (81.8%) and 7 (63.6%) cases, respectively.

## Discussion

A hybrid robotic and laparoscopic approach to the two-stage restorative proctocolectomy allows a safe introduction of the robotic proctectomy, combined with training opportunities and reduced operating time of the more standardized approach of the laparoscopic colectomy. The advantages of robotic surgery, particularly in pelvic dissection and anastomosis construction, cannot be overstated, especially in the narrow pelvis of male or obese patients. The robotic platform offers improved maneuverability and three-dimensional visualization, allowing for more precise work in the confined pelvic space. These ergonomic and technical benefits can facilitate safer and more efficient proctectomy, albeit at the expense of extended operating time, particularly in the learning curve.[1] Moreover, a multiquadrant procedure may not be suitable for surgeons at early stages of the robotic surgery proficiency curve.

Through our analysis of the scientific literature, we found two studies suggesting a hybrid approach combining laparoscopic and robotic techniques for restorative proctocolectomy. Morelli et al.[12] introduced a hybrid approach combining hand-assisted laparoscopic and robotic methods for restorative proctocolectomy in ulcerative colitis patients. The procedure starts with a hand-assisted laparoscopic colectomy via a suprapubic incision, allowing for extracorporeal mobilization of the small bowel and the creation of a tension-free J-pouch. The second phase utilizes the Da Vinci robotic system for a precise proctectomy, focusing on mesorectal plane dissection to preserve pelvic nerves and safely mobilize the rectum down to the sphincters. The final phase involves trans-anal rectal resection, followed by J-pouch insertion and hand-sewn ileal pouch-anal anastomosis (IPAA), concluding with a diverting ileostomy.

Birrer et al.[13] described a hybrid approach using laparoscopy for the subtotal colectomy, followed by robotic completion proctectomy and pouch formation in a separate procedure, as commonly performed in a 3-stage surgery. Our results add to this existing literature for the focus on the introduction of the approach during the robotic surgery learning curve, but also for the attention to the proportion of the procedure performed by surgical trainees. We expect that our approach would result in enhanced training opportunities for both junior trainees, who need training in laparoscopic surgery, as well as more senior trainees or early consultants, who require proctoring on the robotic pelvic dissection.

A growing body of literature compares the efficacy of robotic IPAA to the conventional laparoscopic approach, examining key factors such as complication rates, postoperative outcomes, and operating time. Several studies highlight the similarities between robotic and laparoscopic approaches regarding short-term postoperative outcomes, similarly to our findings. Panteleimonitis et al.[14] reported no statistically significant differences between the two approaches in terms of safety and morbidity, with a trend favoring a shorter length of stay for robotic IPAA. Similarly, Lightner et al.[15] demonstrated equivalent 30-day outcomes for both robotic and laparoscopic IPAA, with no notable differences in the rates of complications such as superficial surgical site infection, peri-pouch abscess, anastomotic leak, pelvic abscess, readmission, or reoperation.

However, Gebhardt et al.[16] noted that while robotic-assisted proctectomy with IPAA is comparable to laparoscopic procedures in terms of short-term clinical outcomes, it is associated with longer operative times and higher surgical costs. This highlights a critical consideration in the adoption of robotic surgery, while technically feasible and safe, the potential benefits of robotics may not immediately translate into substantial short-term clinical advantages over laparoscopy. The hybrid approach we presented demonstrates that the advantages of the robotic platform can be merged with the laparoscopic approach, allowing similar if not shorter operating time.

More recent studies, however, suggest emerging clinical advantages for robotic IPAA, particularly regarding functional outcomes and hospital stay. Hanaoka et al.[17] demonstrated that robotic stapled-IPAA for UC patients resulted in better short-term outcomes and improved defecatory function, especially in cases with lower anastomosis levels compared to laparoscopy. These findings suggest that robotics may confer specific advantages in the preservation of pelvic nerve function, potentially improving postoperative continence and quality of life.

Khawaja et al.[18] conducted a systematic review of studies comparing robotic and laparoscopic approaches in IPAA procedures between 2010 and 2022, encompassing 271 patients who underwent robotic proctectomy with or without colectomy. While the robotic approach was associated with a slightly longer mean operating time, other perioperative outcomes such as mean blood loss, bowel function recovery, mean hospital stay, and conversion rates to open surgery were found to be significantly lower compared to both laparoscopic and open techniques.

Violante et al.[19] conducted a case-matched analysis at a high-volume center and found that robotic IPAA reduced the risk of conversion to open surgery, lowered intraoperative blood loss, and shortened the length of stay compared to laparoscopy. Similarly, Young et al.[20] analyzed outcomes in rectal surgery and reported that hospital stay was significantly longer for patients undergoing laparoscopic procedures (7.5 days) compared to robotic procedures (5.7 days, *P* < 0.01). This difference was even more pronounced when comparing patients who underwent hybrid laparoscopic-assisted total mesorectal excision (TME) with those who underwent robotic TME (8.2 vs. 5.7 days, respectively, *P* < 0.01). Additionally, the conversion rate was 7.9% in the laparoscopic group and 0% in the robotic group (*P* = 0.09).

Additionally, patient factors play a significant role in postoperative outcomes. Obese patients undergoing total proctocolectomy or completion proctectomy with IPAA have an increased risk of morbidity. Furthermore, patients receiving steroid or immunosuppressive therapy preoperatively should ideally delay surgery or be more appropriately treated with a 3-stage approach to mitigate surgical risks.[21]

The cost-effectiveness of robotic versus laparoscopic rectal surgery remains a subject of debate. While robotic rectal resection is associated with higher direct costs in the short term, evidence suggests that it provides better perioperative outcomes, including reduced blood loss, lower postoperative pain, shorter hospital stays, and fewer readmissions, ultimately leading to improved quality-adjusted life years (QALYs).[22] However, other analyses indicate that despite its technological advantages, robotic rectal surgery remains significantly more expensive than laparoscopy, without demonstrating a clear superiority in oncological outcomes, complication rates, or long-term quality of life.[23] These findings highlight the need for further research to define the most appropriate indications for robotic surgery, balancing clinical benefits with cost considerations to ensure sustainable implementation in routine practice.

When comparing robotic and laparoscopic techniques for ileal pouch-anal anastomosis (IPAA), several studies have consistently reported longer operating times for robotic procedures. According to a systematic review by Flynn et al.[24] which included 640 patients undergoing either robotic, laparoscopic, or open proctectomy with IPAA, robotic surgery was associated with significantly longer operative times. Across various studies, mean operating times for robotic proctectomy and IPAA ranged from 247 to 407 min, while laparoscopic cases averaged between 234 and 316 min.

These prolonged operating times were largely attributed to the additional complexity of robotic system setup and the learning curve associated with mastering the robotic platform. This finding was statistically significant in all studies, suggesting that despite the technical advantages of robotic surgery, time efficiency remains a challenge.

One factor that may account for variability in outcomes between robotic and laparoscopic IPAA is the surgeon’s learning curve. Many studies comparing these techniques were conducted during the initial or early experience of surgeons with robotic surgery. In most studies, the authors describe their robotic experience as part of the learning phase, where surgeons were highly experienced in colorectal surgery but had limited exposure to robotics.[25, 26] While robotic proctectomy for inflammatory bowel disease (IBD) was feasible, its clinical advantages may only become apparent once surgeons overcome the learning curve and variability in outcomes may stem from the surgeon’s level of expertise with robotic platforms.

When comparing operative times in total robotic proctocolectomy performed in 2 stages, it is crucial to consider the experience of the surgical team, as highlighted in the study by Bianchi et al.[27] In their single-center experience, the mean operative time for total robotic proctocolectomy (TPC) in 16 patients was reported as 271.42 min. Although this time is relatively short, it is important to recognize that the procedures were performed by a highly experienced robotic surgery team. As such, while these results are valuable for comparison, they may not be directly applicable to all centers.

In contrast, the work by Hollandsworth et al.[28] provides a more comprehensive analysis of the impact of the learning curve on operative times for total proctocolectomy with the da Vinci Xi system. Their findings show a clear trend of improvement over time. Initially, in 2016, the mean operative time was significantly longer at 498 min, reflecting the challenges faced by surgeons during the early stages of the learning curve. However, by 2018, the mean operative time had decreased to 227 min, underscoring the potential for significant time savings as surgeons gain proficiency with robotic techniques. This progression highlights how, with experience, robotic proctocolectomy becomes more efficient.

Given these observations, our proposed hybrid approach—combining robotic and laparoscopic techniques—offers an attractive alternative, particularly for surgeons in the early stages of their robotic surgery learning curve. By incorporating key laparoscopic steps, the hybrid technique allows for reduced operative time without sacrificing the precision and advantages of robotic technology. We anticipate that this hybrid approach can facilitate smoother transitions for less experienced surgeons, ultimately leading to shorter operative times and improved outcomes as the learning curve is navigated.

Furthermore, in recent years, new robotic platforms have emerged alongside the Da Vinci system,

with unique features influencing surgical workflow, docking strategies, and learning curves. The Medtronic Hugo™ RAS system, compared to conventional robotic platforms, offers modular robotic arms and an open console design, allowing for flexible docking configurations that can be adapted to different surgical needs.

A recent study[29] comparing robotic-assisted and laparoscopic surgery for inflammatory bowel disease (IBD) demonstrated that robotic surgery using the Hugo™ RAS system is safe and feasible, with comparable postoperative outcomes to laparoscopy. Although the robotic approach was associated with a longer operative time, it allowed for a higher rate of intracorporeal anastomosis, which may provide long-term benefits in minimally invasive colorectal surgery. Importantly, conversion rates and postoperative complications, including intra-abdominal septic complications, ileus, and bleeding, were similar between the two approaches.

However, it appears that with these newer platforms, the redocking process may be more complex and could result in significant time loss. This is an additional factor that must be considered when evaluating the overall efficiency of robotic surgery. Recently, a dedicated study[30] evaluating a refined multidocking strategy for robotic low anterior resection (LAR) with the Hugo™ RAS system highlighted its efficiency and adaptability. The study demonstrated a significant reduction in docking times as surgeons gained experience, with proficiency reached after approximately 15 procedures. This suggests that tailored docking strategies can optimize the utilization of modular robotic platforms, potentially easing the transition from laparoscopy to robotics.

The hybrid technique that we propose may lead to a reduction in operative time, a factor that could further enhance its appeal. The ability to maintain efficiency while providing high-quality training and minimizing technical challenges makes this approach a promising option in the field of restorative proctocolectomy.

Performing a fully robotic total colectomy can be a time-consuming and technically demanding task, often requiring hours in the operating room for those at the start of their robotic training. By isolating the robotic approach to a specific, more standardized portion of the procedure, this technique allows surgeons to focus their efforts and refine their skills in a more controlled manner. This not only accelerates the learning process but also fosters greater consistency in outcomes.

Moreover, the structured stepwise approach, with distinct phases of varying complexity, offers an ideal balance between precision and skill acquisition. The most challenging portion, the robotic phase, is managed by the lead consultant or an experienced surgeon, ensuring the highest level of expertise where it is most needed. At the same time, the laparoscopic phases can be performed by surgeons in training or fellows, providing them with valuable hands-on experience without compromising the overall success of the operation. This division of tasks helps address a common concern with robotic surgery—the potential hindrance in developing core laparoscopic skills in younger surgeons—by ensuring they remain actively involved in key parts of the procedure. We acknowledge that our results are limited by the small sample size and the lack of a completely robotic proctocolectomy group. This is mainly due to most of the restorative procto-colectomies being performed in 3 stages in our center. However, our results compare with the existing literature on robotic IPAA. A longer follow-up will also allow us to report on patient-reported outcome measures.

In conclusion, this hybrid technique leverages the precision of robotic surgery and the versatility of laparoscopy, offering a minimally invasive yet highly effective solution for complex cases of ulcerative colitis requiring proctocolectomy with IPAA.

## Data Availability

The data that support the findings of this study are not openly available due to reasons of sensitivity and are available from the corresponding author upon reasonable request. Data are located in controlled access data storage at Chelsea and Westminster Hospital NHS Foundation Trust, London, UK.
